# Identification in GRMD dog muscle of critical miRNAs involved in pathophysiology and effects associated with MuStem cell transplantation

**DOI:** 10.1186/s12891-016-1060-5

**Published:** 2016-05-11

**Authors:** Florence Robriquet, Candice Babarit, Thibaut Larcher, Laurence Dubreil, Mireille Ledevin, Hélicia Goubin, Karl Rouger, Laëtitia Guével

**Affiliations:** INRA UMR 703 PAnTher “Physiopathologie Animale et bioThérapie du muscle et du système nerveux”, Oniris, Atlanpôle - La Chantrerie, Route du Gachet C.S. 40706, F-44307 Nantes Cedex 03, France; LUNAM Université, Oniris, École nationale vétérinaire, agro-alimentaire et de l’alimentation Nantes-Atlantique, F-44307 Nantes, France; Université de Nantes, F-44322 Nantes, France

**Keywords:** miRNA, Stem cell therapy, Duchenne muscular dystrophy, GRMD dog, Skeletal muscle, *In situ* hybridization

## Abstract

**Background:**

Duchenne muscular dystrophy (DMD) is an X-linked muscle disease that leads to fibre necrosis and progressive paralysis. At present, DMD remains a lethal disease without any effective treatment, requiring a better understanding of the pathophysiological processes and comprehensive assessment of the newly identified therapeutic strategies. MicroRNAs including members of the muscle-specific myomiR family have been identified as being deregulated in muscle of DMD patients and in *mdx* mice used as a model for DMD. In recent years, the Golden Retriever muscular dystrophy (GRMD) dog has appeared as the crucial animal model for objectively assessing the potential of new innovative approaches. Here, we first aim at establishing the muscle expression pattern of five selected miRNAs in this clinically relevant model to determine if they are similarly affected compared with other DMD contexts. Second, we attempt to show whether these miRNAs could be impacted by the systemic delivery of a promising stem cell candidate (referred to as MuStem cells) to implement our knowledge on its mode of action and/or identify markers associated with cell therapy efficacy.

**Methods:**

A comparative study of miRNAs expression levels and cellular localization was performed on 9-month-old healthy dogs, as well as on three sub-sets of GRMD dog (without immunosuppression or cell transplantation, with continuous immunosuppressive regimen and with MuStem cell transplantation under immunosuppression), using RT-qPCR and *in situ* hybridization.

**Results:**

We find that miR-222 expression is markedly up-regulated in GRMD dog muscle compared to healthy dog, while miR-486 tends to be down-expressed. Intriguingly, the expression of miR-1, miR-133a and miR-206 does not change. *In situ* hybridization exploration reveals, for the first time, that miR-486 and miR-206 are mainly localized in newly regenerated fibres in GRMD dog muscle. In addition, we show that cyclosporine-based immunosuppression, classically used in allogeneic cell transplantation, exclusively impacts the miR-206 expression. Finally, we demonstrate that intra-arterial administration of MuStem cells results in up-regulation of miR-133a and miR-222 concomitantly with a down-expression of two sarcomeric proteins corresponding to miR-222 targets.

**Conclusion:**

We point out a differential muscle expression of miR-222 and miR-486 associated with the pathophysiology of the clinically relevant GRMD dog model with a tissue localization focused on regenerated fibres. We also establish a modified expression of miR-133a and miR-222 subsequent to MuStem cell infusion.

**Electronic supplementary material:**

The online version of this article (doi:10.1186/s12891-016-1060-5) contains supplementary material, which is available to authorized users.

## Background

Duchenne muscular dystrophy (DMD) is a progressive and fatal X-linked recessive disorder of skeletal and cardiac muscles. It is a particularly severe and common form of muscular dystrophy, affecting one in 3500 males at birth [1]. Mutations in the gene encoding the dystrophin lead to a lack of this protein, which normally ensures the essential link between the subsarcolemmal cytoskeleton and the extracellular matrix at the muscle fibre membrane [2, 3]. DMD is characterized by repeated cycles of necrosis/regeneration of muscle fibres, progressive replacement of skeletal muscle by fibrotic and adipose tissues and generalized muscle weakness, paralysis and death [4]. Recently, several gene and cell-based strategies have been developed to restore dystrophin expression in the Golden Retriever muscular dystrophy (GRMD) dog, the clinically relevant animal model of DMD. Some of these innovative approaches have now entered preclinical studies [5, 6]. In parallel, numerous studies are ongoing to define muscle molecular signatures that could be used to characterize dystrophic dog tissue [7, 8] or to validate the effect of promising therapeutic strategies [9, 10].

MicroRNAs (miRNAs) are short non-coding RNA sequences of 21 to 25 nucleotides that regulate gene expression at a post-transcriptional level. Through binding to target mRNA, they promote its degradation or translational inhibition [11, 12]. In muscle, specific miRNAs (known as myomiRs), such as miR-1, miR-133 and miR-206, are involved in regulation of the proliferation or differentiation of myogenic cells [13–16] and are especially regulated by transcription factors implicated in muscle growth and development [17, 18]. Other miRNAs, such as miR-29, miR-34, miR-222 and miR-486, also play key-roles in modulating important pathways of skeletal muscle processes [19–22]. Over the last few years, miRNAs have been found to be deregulated in muscular dystrophies [23, 24]. A specific DMD signature has been identified based on eleven miRNAs that are deregulated both in *mdx* mice and DMD patients [22]. As regards myomiRs, several studies report that miR-1 and miR-133 are under-expressed, while miR-206 is over-expressed in *mdx* muscles [25–27]. All these findings indicate an important role of miRNAs in pathophysiological pathways regulating muscle response to damage and regeneration. However, except for a preliminary study performed on *CXMDJ* dog muscle [26], there is currently no experimental data concerning miRNA status specifically in GRMD dog skeletal muscle. Alternatively, a recent study identified deregulated miRNAs in the serum of GRMD dogs [28]. Although GRMD dogs more closely mimic the human disease than *mdx* mouse, the lack of data on this large animal model represents a real limitation for the accurate description of the dysregulation of miRNAs in a DMD-like context. Moreover, it is important to fill this gap in our knowledge of the GRMD dog model, in particular with regard to the preclinical evaluation of new therapeutic proposals.

We show that systemic delivery of MuStem cells (which are muscle-resident stem cells isolated from healthy dog based on delayed adhesion properties) represents an attractive avenue for future therapeutic applications in DMD patients. Indeed, allogeneic MuStem cell transplantation in GRMD dogs leads to reduced muscle damage, increased regeneration activity, and a persistent stabilization of clinical status [29]. In a previous study, we revealed the impact of MuStem cell transplantation, with an up-regulation of genes reflecting a sustained enhancement of muscle regeneration [30]. In addition, MuStem cells can act on several other biological pathways implicated in protein degradation mechanisms and energy metabolism, evoking a diffuse impact with a large number of targeted biological processes.

In the present study, we firstly aim at defining the miRNA pattern in the skeletal muscle of 9-month-old GRMD dogs corresponding to an advanced state of the disease. Secondly, we attempt to determine how this pattern could be affected following the intravenous delivery of MuStem cells. We determine, for the first time, that miR-222 displays a differential expression pattern in GRMD dog muscle as shown by its marked up-regulation. Using *in situ* hybridization, we show that miR-206 and miR-486 are mainly expressed in clusters of newly regenerated fibres. In addition, we demonstrate an up-regulation of both miR-133 and miR-222 4 months after MuStem cell transplantation, highlighting their potential use as novel markers for the follow-up of effects associated with MuStem cell delivery in a dystrophic context.

## Methods

### Ethics statement and animals

This study was approved by the Ethics Committee on Animal Experimentation of the Pays de la Loire Region (France) in accordance with the guidelines from the French National Research Council for the Care and Use of Laboratory Animals (Permit Number: CEEA.2012.104). All the dogs were obtained from the Centre d'élevage du Domaine des Souches (Mézilles, France), which were kept at the Boisbonne Centre for gene and cell therapy of Oniris (Nantes, France). Fourteen 9-month-old golden retriever dogs were included in the study; five were healthy and nine were GRMD. Affected dogs were identified in the 1st week of life using polymerase chain reaction (PCR)-based genotyping. This identification was corroborated by a dramatic and early rise in levels of serum creatine kinase [31]. GRMD dogs were divided into three subsets: three GRMD dogs received neither an immunosuppressive regimen nor cell transplantation (subset denoted as GRMD), three received only a continuous immunosuppressive regimen (mock GRMD) and the remaining three received MuStem cell transplantation under immunosuppression (GRMD^MuStem^) (Table 1).

**Table 1:** Description of the fourteen male dogs included in the miRNA study

Study	Dog group	Number of animals	Phenotype	Immunosuppression	MuStem cell injection
Physiopathology	Healthy	5	healthy	None	None
	GRMD	3	dystrophic	None	None
MuStem cell impact	mock GRMD	3	dystrophic	Yes	None
	GRMD^MuStem^	3	dystrophic	Yes	Yes

### Cell delivery procedure

MuStem cells were isolated from a pool of hindlimb muscles of 2.5-month-old healthy dogs and prepared as previously described [29]. Three GRMD dogs (ranging from 3.5 to 4.5 months of age) were submitted to a continuous cyclosporine-based immunosuppressive regimen as established by Rouger et al. (2011) and received three cell injections (spaced at an interval of 2 weeks): GRMD^MuStem^. Each of these injections delivered 5.5×10^7^ to 8.0×10^8^ MuStem cells/kg into the cephalic vein using laminar flow at a rate of 12 mL/min.

### Clinical follow-up

A clinical score was measured weekly for all GRMD dogs following a previously described method [29, 32]. Dogs were weighted and clinically assessed in a non-blinded manner by a veterinarian on a weekly basis during all the experiment. Briefly, this clinical score is based on 11 locomotion and muscular criteria and 6 items related to general health status. It is expressed as a percentage of the maximum score defined as 100 % for a healthy dog.

### Muscle sampling

*Biceps femoris* muscle samples (0.5 cm^3^ fragments) were collected surgically from the middle portion of the muscle in 9-month-old (37 ± 5-week-old) healthy, GRMD, mock GRMD and GRMD^MuStem^ dogs. The *Biceps femoris* is a large and easily accessible muscle. This time-point corresponds to 4 months after systemic administration to the GRMD^MuStem^ dogs and is the same as in our previous transcriptomic study [30]. Muscle fragments were divided into two parts for histological and molecular analyses, and subsequently stored at −80 °C until processing.

### Histological analysis

Eight μm-thick cryosections were incubated with mouse primary antibody directed against the developmental myosin heavy chain (MyHCd, 1/20, Novocastra, Newcastle, United Kingdom) for 1 h at 37 °C. After successive incubation with a secondary biotinylated antibody and streptavidin horseradish peroxidase conjugate (1/300, Dako, Glostrup, Denmark), MyHCd protein was visualized by diamidinobenzidine tetrahydrochloride (DAB; Dako). Slides were then dehydrated and mounted in a dry mounting medium. Morphometric analysis was performed using a digital camera (Nikon DXM 1200, Nikon Instruments, Badhoevedorp, The Netherlands) combined with image-analysis software (NIS, Nikon). Microscopic fields were randomly selected on immunolabelled sections using intermediate magnification to observe at least 100 fibres. To determine the percentage of MyHCd^+^ fibres, at least 662 fibres (1030 ± 125) were counted on three randomly selected microscopic fields. For each measurement, reproducibility is better than 92 %. For dystrophin labelling, all acquisitions were performed with the same signal amplification resulting from identical detector gain, as previously described [29]. To determine the proportion of dystrophin^+^ fibres, 880 ± 101 total fibres were counted (laminin red fluorescent immunolabelling) in the *Biceps femoris m*uscle sections of the GRMD^MuStem^ dogs (*n* = 3) and then the number of fibres expressing dystrophin was determined from DYS2 (Novocastra) green fluorescent immunolabelling.

### miRNAs isolation and qPCR

miRNAs were extracted from muscle samples of right and left *Biceps femoris* of each animal. The mirVana miRNA isolation kit (Ambion, Austin, TX, USA) was used, according to the manufacturer’s instructions, and microRNAs were finally eluted with 100 μL of water and quantified using a Nanodrop spectrophotometer (Labtech, Wilmington Delaware, USA). Reverse Transcription reactions were carried out on 5 ng of miRNAs using the TaqMan miRNA Reverse Transcription kit (Applied Biosystems, Foster City, CA, USA) and miRNA-specific stem loop primers for miR-1, miR-133a, miR-206, miR-222, and miR-486 (Applied Biosystems miRNA assays). Real-time PCR reactions were performed at least in duplicate with miRNA-specific primers and Taqman® probes on the CFX96 PCR System (BioRad). Data were normalized using U6 snRNA (RNU6B) as an internal control and differential expression was calculated using the 2^-∆∆Ct^ method. For each miRNA, statistical differences between two groups were analysed by a Mann–Whitney test.

### *In situ* hybridization

*In situ* hybridization (ISH) was performed on muscles from healthy, GRMD, mock GRMD and GRMD^MuStem^ dogs, using digoxigenin (DIG)-labelled miRCURY locked nucleic acid (LNA) detection probes (Exiqon, Vedbaek, Denmark), corresponding to hsa-miR-486 (38596–05), hsa-miR-206 (88081–15) and scramble-miR (99004–05 and 99004–15). Ten μm-thick frozen muscle sections were air-dried for 1 h and fixed for 10 min in 4 % paraformaldehyde. Then, the sections were permeabilized with proteinase K (20 μg/mL) for 10 min. For pre-hybridization, the tissue sections were covered for 1 h with hybridization buffer containing 50 % formamide, 4X SSC, 1X Denhardt’s solution, 500 μg/mL salmon sperm DNA (Sigma-Aldrich, Saint Quentin Fallavier, France), 10 % dextran sulfate and 1X Blocking Reagent (Roche, Basel, Switzerland). For hybridization, 50 nM of DIG-labelled probes diluted in hybridization buffer were applied per section and incubated in a sealed humidified chamber for 16 h at 55 °C. A stringency wash was performed for 30 min in 50 % formamide/1X SSC, followed by two 0.2X SSC washes for 15 min each. Sections were then incubated with alkaline phosphatase-conjugated sheep anti-DIG (1/1000, Roche) antibody for 2 h. Hybridized probes were visualized by color reaction with nitro-blue tetrazolium (NBT) and 5-bromo 5-chloro-3-indolyl phosphate (BCIP) overnight at 4 °C. Slides were counterstained with Nuclear Fast Red and mounted in a Vectamount mounting medium (Vector Laboratories, Burlingame, USA). *In situ* analysis was carried out by one “blinded” reader and one non-“blinded” reader, yielding comparable results. No signal was detected using scrambled control probes.

### Western Blot

For protein extraction, muscles were homogenized in RIPA lysis buffer containing 150 mM NaCl, 50 mM Tris–HCl pH 7.4, 1 % Nonidet-P40, 1 % glycerol, 1 mM EDTA and protease inhibitors using the Precellys (Ozyme, France) (2 × 10 s, 6500 rpm). Homogenates were centrifuged at 10,000 g to pellet debris and supernatants were centrifuged at 20,000 g (45 min, 4 °C). Protein concentration was determined using a BCA protein assay (Sigma-Aldrich). Fifty μg of proteins of tissue homogenate were resolved by sodium dodecyl sulfate polyacrylamide gel electrophoresis (SDS-PAGE) on 4–12 % polyacrylamide gels (NuPage, Life Technologies, Illkirch, France) and electroblotted onto nitrocellulose membranes (Protran BA 83, GE Healthcare) using a Bio-Rad® liquid blotting system at 30 mA for 2 h. The membranes were blocked using 50 % Blocking Buffer (Odyssey®, Li-Cor Biosciences, Lincoln, NE, USA) in PBS (1 h, room temperature) and incubated overnight at 4 °C with primary antibodies against myosin heavy chain (MHC) MF-20 (1/1000, Developmental Studies Hybridoma Bank /DSHB, Iowa City, IA, USA), α-actinin (1/1000, Sigma-Aldrich), MYH7 (1/5000, Abcam, Cambridge, MA, USA). After washing with Tween 0.1 % in PBS, the blots were incubated with horseradish peroxidase-conjugated or fluorophore-conjugated anti-mouse and anti-rabbit secondary antibody. After washing, the samples were coverslipped with Mowiol Mounting Medium (Calbiochem EMD Biosciences, San Diego, CA, USA) and scanned with a blue 488 nm argon ion laser using the C1 inverted Nikon TE-2000 laser scanning confocal microscope (Nikon, Champigny, France). Equal protein loading was checked through α-actinin labelling and Ponceau S staining of the membranes.

## Results

### Differential miRNA expression level in GRMD dog muscle

**Fig. 1:**
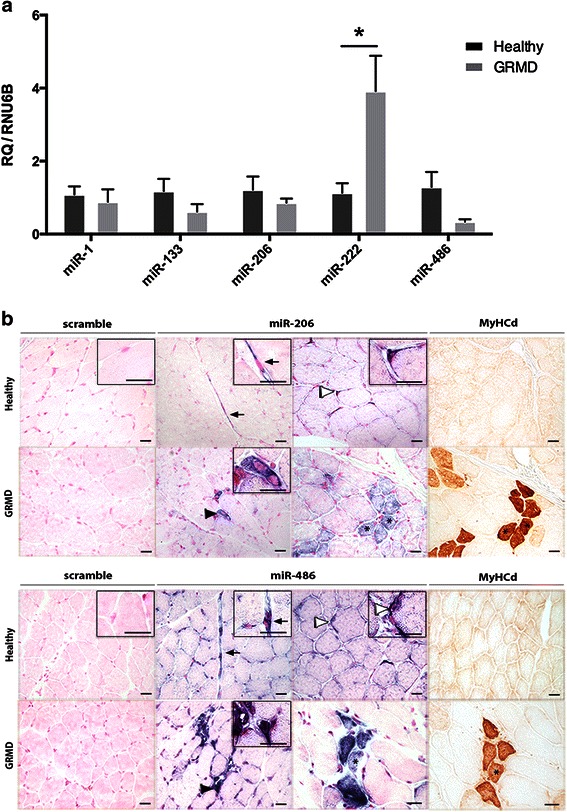
**a** Relative expression levels of miRNAs in dog muscle. Expression levels of miR-1, miR-133a, miR-206, miR-222 and miR-486 were determined in 9-month-old healthy (*n* = 5) and GRMD (*n* = 3) dog muscle by real-time PCR and were normalized to RNU6B levels. Results are indicated as relative expression and are presented as mean ± SEM. *p < 0.05. **b** miR-206 and miR-486 localization in healthy and GRMD dog skeletal muscle. The tissue localization was assayed by *in situ* hybridization on *Biceps femoris* muscle cryosections derived from healthy and GRMD dogs, using digoxigenin labelled LNA probes. Representative images are shown. Upper panel: miR-206 expression. In healthy dog muscle, miR-206 expression is detected in cytoplasmic processes of vessel endothelial cells (*black arrow*) and around some peripheral nuclei (*empty arrow head*) of muscle fibres. In GRMD dog muscle, a strong signal is detected in myoblasts (*black arrow head*) and regenerating MyHCd^+^ fibres (*asterisk*). Lower panel: miR-486 expression. In healthy dog muscle, miR-486 is detected both in endothelial cells (*black arrow*) and around peripheral nuclei (*empty arrow head*). In GRMD dog muscle, miR-486 is localized in myoblasts (*black arrow head*) and regenerating fibres (*asterisk*). Scale bar = 25 μm

The expression levels of five miRNAs in healthy and GRMD dog muscles were investigated to determine whether miRNAs (previously shown to be differentially expressed in skeletal muscle of DMD patients and *mdx* mice) display the same deregulations in the clinically relevant dog model. The generated data are normalized to RNU6B, which is an internal control RNA frequently used in miRNA expression studies, yielding an average Ct of 30.89 ± 0.93 (SD) for the whole set of tested samples (Additional file 1: Fig. S1). In the dystrophic context, we observe a markedly increased expression of miR-222 (*p* = 0.03) and a tendency to decreased expression of miR-486 (*p* = 0.14) (Fig. 1a). On the other hand, miR-1, miR-133a, miR-206 expression levels are unchanged in GRMD dogs.

### miRNA cellular localization in GRMD dog muscle

To complete our PCR-based observations, we investigated the subcellular and tissue localization of the miRNAs in a dystrophic context by using ISH. We successfully performed *in situ* exploration of two of the five miRNA tested: miR-206, a key modulator of skeletal muscle development and disease, and miR-486, an important factor in the regulation of DMD muscle pathology (Fig. 1b). In healthy dog muscle, miR-206 and miR-486 are expressed around some peripheral nuclei of fibres as well as in endothelial cells. Furthermore, most of the fibres display a perinuclear miR-486 signal. Interestingly, in GRMD dogs, miR-206 and miR-486 are mainly localized in newly formed myoblasts and regenerating fibres, as demonstrated by MyHCd^+^ labelling on serial sections. Unfortunately, we failed to detect significant signal for miR-222 and miR-133a.

### Clinical and tissue consequences of the intravenous delivery of MuStem cells

**Fig. 2:**
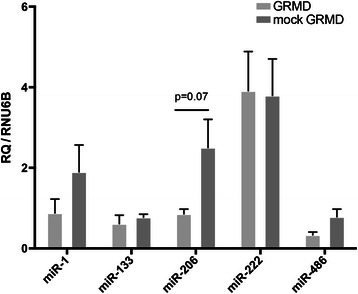
Relative expression levels of miRNAs in GRMD dog muscle under immunosuppressive regimen. Expression levels of miR-1, miR-133a, miR-206, miR-222 and miR-486 were determined in muscles (*right* and *left* *Biceps femoris*) of three 9-month-old GRMD and six mock GRMD dog by real-time PCR and normalized by RNU6B levels. Results are indicated as relative expression and are presented as mean ± SEM

In the context of the allogeneic transplantation protocol, GRMD^MuStem^ dogs and mock GRMD dogs were both submitted to immunosuppression. To determine whether cyclosporine treatment on its own can modify the miRNA profile, the expression levels were first determined in GRMD dogs with or without immunosuppression. We observe no significant change except for miR-206, which is increased under our immunosuppressive regimen (*p* = 0.07) (Fig. 2). Interestingly, even though miR-206 expression is modified, its subcellular localization remains unchanged (data not shown here).

**Fig. 3:**
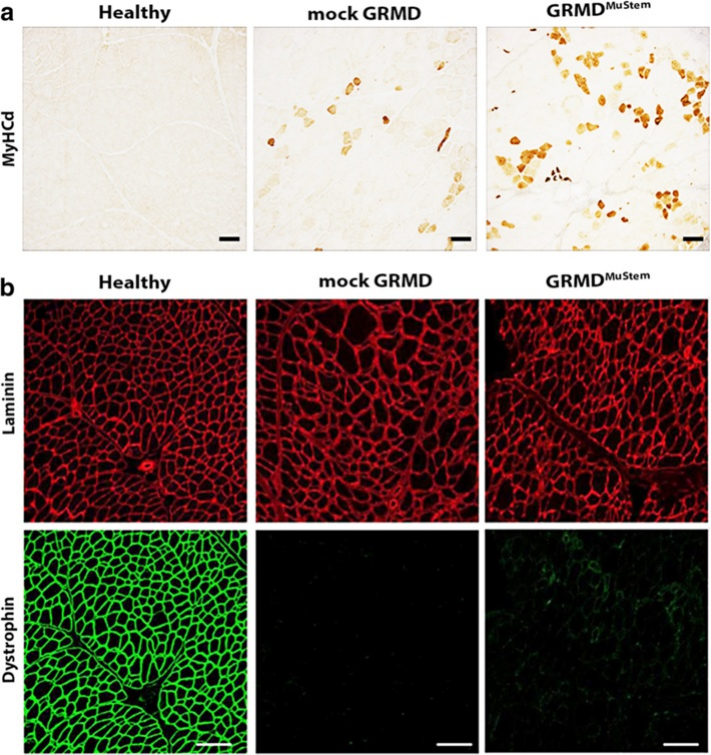
**a** MyHCd immunolabelling in healthy, mock GRMD and GRMD^MuStem^ dogs. Transverse cryosections of the *Biceps femoris* muscle of 9-month-old healthy, mock GRMD and GRMD^MuStem^ dogs. Scale bar = 50 μm. **b** Dystrophin immunolabelling in healthy, GRMD and GRMD^MuStem^ dogs. Transverse cryosections of the *Biceps femoris* muscle of 9-month-old healthy, mock GRMD and GRMD^MuStem^ dogs. The laminin (*red*) and dystrophin (*green*) fluorescent immunolabellings are presented. Scale bar = 200 μm

Mock GRMD dogs display a progressive clinical impairment characterized at 9 months age by a clinical score of 43.5 % ± 25.2. By contrast, all GRMD^MuStem^ dogs display an early and persistent stabilization of their clinical score that is maintained above 80.9 % ± 9.3 at the same age. Repeated-measures ANOVA carried out from 7.5 to 8.5 months of age indicates a trend towards a positive effect of the MuStem cell delivery (F = 3926; *p* = 0.059) (Additional file 2: Fig. S2). Regenerative activity is also assessed on muscle sections using a specific MyHCd labelling. While 12.9 % ± 5.3 of the fibres express this developmental isoform in mock GRMD dog muscle, the proportion of MyHCd^+^ fibres is 20.5 % ± 3.9 in GRMD^MuStem^ dogs, thus demonstrating a tendency to an increased muscle regenerative activity following MuStem cell delivery (Fig. 3a).

Immunofluorescent labelling of dystrophin in the *Biceps femoris* muscle shows no expression of this protein in mock GRMD dogs, apart from some rare positive fibres corresponding to revertant fibres. Muscle sections of GRMD^MuStem^ dogs are characterized by a very low expression level of dystrophin compared to that observed in healthy dog muscle (Fig. 3b). Fibres observed in healthy dog are defined by a high fluorescent labelling intensity as well as by continuous labelling all along the fibre membrane. On the contrary, the limited dystrophin expression observed in GRMD^MuStem^ dog muscle is illustrated by a discontinuous labelling along the membrane and is restricted at the most to 14.4 % of all fibres (Fig. 3b).

### Modified muscle miRNA expression following MuStem cell transplantation

**Fig. 4:**
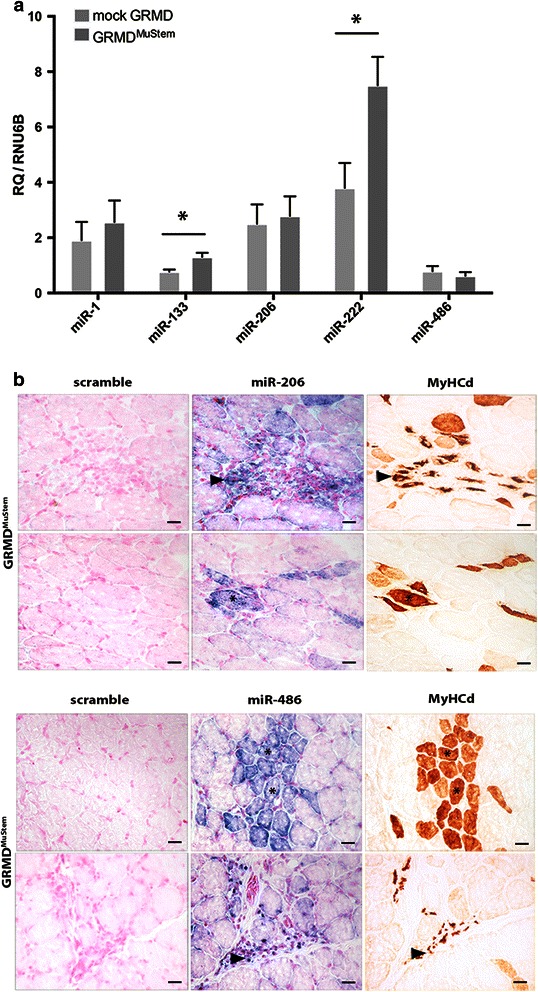
**a** Modulation of miRNA levels in GRMD dog muscles after systemic administration of MuStem cells. Expression levels of miR-1, miR-133a, miR-206, miR-222, and miR-486 were determined in muscles (*right* and *left* *Biceps femoris*) of six 9-month-old GRMD^MuStem^ dogs compared to six mock GRMD dogs. RNU6B was used as internal control and relative expressions are presented as mean ± SEM. *p < 0.05. **b** miRNA localization in GRMD dog muscle after MuStem cell transplantation. Upper panel: miR-206 expression. Lower panel: miR-486 expression. miR-206 and miR-486 are detected in myoblasts (*black arrow head*) and in the cytoplasm of small and intermediate regenerating MyHCd^+^ fibres (*asterisk*). Scale bar = 25 μm

**Fig. 5:**
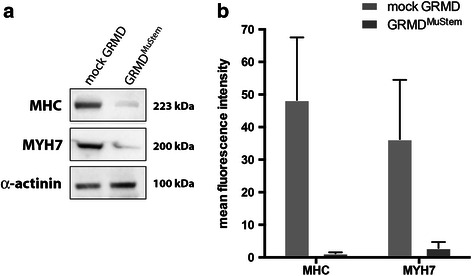
**a** MHC and MYH7 protein expressions in mock GRMD compared to GRMD dog after MuStem cell transplantation. **a** Representative western blot analyses. α-actinin is used as control. **b** Graphical representation of immunoblot analyses in three mock GRMD and GRMD^MuStem^ samples. The mean fluorescence intensity is represented along with the SEM (standard error of the mean) for the different samples

The expression level of five miRNAs was investigated on the GRMD^MuStem^ dogs to determine whether cell transplantation could have an impact on their expression. We show that miR-133a (*p* = 0.03) and miR-222 (*p* = 0.03) are up-regulated in GRMD^MuStem^ dogs compared to mock GRMD dogs (Fig. 4a), while miR-1, miR-206 and miR-486 expressions appear unchanged. In terms of tissue distribution, the ISH carried out on the *Biceps femoris* muscle of GRMD^MuStem^ dog reveals an expression of miR-486 and miR-206 on clustered MyHCd^+^ regenerative fibres (Fig. 4b), which confirms previous findings [33, 34] and reinforces the hypothesis whereby these two miRNAs are unaffected by the infusion of cells. Lastly, while miR-222 is up-regulated in GRMD versus healthy dog muscle, it is found to be even more up-regulated in GRMD^MuStem^ versus mock GRMD dog muscle (*p* = 0.03) (Fig. 4a). To corroborate this result, we investigated the protein abundance of miR-222 targets (sarcomeric proteins: myosin heavy chain (MHC) and MYH7). We observe a decrease in sarcomeric myosin heavy chain proteins expression in GRMD^MuStem^ dog, reflecting the inhibition of sarcomeric protein accumulation concordant with miR-222 overexpression [20] (Fig. 5a and b).

## Discussion

miRNAs are considered as integral components of the regulatory circuitry for myogenesis, even if their full role in muscle growth and development remains to be elucidated [13–15, 18]. Numerous studies provide increasing evidence for the involvement of miRNAs in myopathies, and particularly in muscular dystrophies [22, 23, 35]. It has been recently reported that miRNAs are promising biomarkers for monitoring disease progression [28, 36, 37]. In this regard, several serum miRNAs have been identified as dysregulated in GRMD dogs, using a high-throughput miRNA sequencing screening [28]. Based on the clinical relevance of the animal model used, these results have allowed authors to select these miRNAs as candidate biomarkers for DMD patients. In addition, it is increasingly acknowledged that miRNAs could represent useful tools in the assessment of experimental therapies to cure muscle diseases [25, 28, 38]. Nevertheless, further investigations need to be conducted to identify the role of these dysregulated miRNAs in muscle pathophysiology.

Up to now, most of the results presented on muscle miRNAs have been obtained from the *mdx* mouse model, which is known to show limitations for the study of pathogenetic mechanisms and therapeutic trials specific to DMD. For this reason, we aim at establishing, for the first time, a description of miRNA dysregulations in GRMD dog skeletal muscle based on a dedicated set: miR-1, miR-133a, miR-206, miR-222 and miR-486. In accordance with previous observations made in the *mdx* mouse model and DMD patients [10, 22, 25, 26], we find that miR-222 and miR-486 exhibit a marked up-regulation and a down-regulation in 9-month-old GRMD dog muscle, respectively. On the contrary, RT-qPCR performed on *Biceps femoris* muscle extract fails to reveal any dysregulation of miR-206, in contrast with the previously described up-regulation in both the *mdx* mouse model and DMD patients [10, 22, 25, 26]. Nevertheless, up-regulation of miR-206 is not observed in all dystrophic muscles. Indeed, McCarthy et al. demonstrated that miR-206 is overexpressed in the most severely affected *mdx* muscles, i.e. the diaphragm, but not in the hindlimb [27]. In addition, Yuasa et al. showed a decreased expression of this miRNA in the *CXMDJ* tibialis anterior muscle compared to the control [26]. Our results support Yuasa’s hypothesis that increased expression of miR-206 in *mdx* muscle may reflect active and efficient regeneration, whereas its decreased expression in *CXMDJ* muscle may illustrate relatively exhausted regeneration potential [26]. In the present study, we use *in situ* detection to obtain original information concerning the muscle tissue distribution of the miRNAs, thus improving the characterization of their tissue expression. Combined *in situ* hybridization and MyHCd labelling demonstrate that miR-206 is histologically related to muscle fibre regenerative processes in GRMD dog, being mainly expressed in newly formed fibres. Interestingly, this distribution has been previously reported in *mdx* muscles [10, 26] that have considerable regenerative capacity [39, 40].

While we describe here the expression patterns of a miRNA subset, further studies are required to understand the implication of miRNAs in the pathophysiology of GRMD dog. In this paper, we show that a continuous cyclosporine-based immunosuppressive regimen maintained over a period of 5 months does not lead to a major modification of the investigated miRNAs levels, except for miR-206 that tends to increase. This result highlights a selective impact of immunosuppression treatment on the expression levels of miRNAs, thus strongly suggesting that the immunosuppressive component must be considered in the assessment of allogeneic cell-based preclinical studies requiring the use of immunosuppression [41].

In the present study, we attempt to determine whether the systemic delivery of MuStem cells, which increases muscle regenerative activity and stabilizes the clinical status of GRMD dogs [29], can concomitantly affect the expression levels of miRNAs that are able to modulate key cellular processes at a post-transcriptional level. This hypothesis seems particularly interesting because the observed clinical and tissue benefits following MuStem cell infusion are linked to a low dystrophin protein level as well as a limited percentage of dystrophin-positive fibres, clearly evoking the implication of other molecular pathways [29, 30]. Firstly, it is surprising that the expression levels of miR-206 and miR-486 (two miRNAs known to be implicated in the regenerative process) are not up-regulated in transplanted GRMD dogs. It could be hypothesized that the increased regenerative potential revealed in GRMD^MuStem^ dogs 4 to 5 months after transplantation is not sufficient to be associated with a differential expression of miR-206. Secondly, we demonstrate an up-regulation of miR-133a and miR-222 expression after systemic delivery of MuStem cells. Interestingly, changes of these miRNAs are reported to be implicated in the disruption of sarcomere organization [20, 42]. Expression of miRNA-222 in myoblasts induces myogenin expression followed by inhibition of sarcomeric protein accumulation. Our finding on the down-expression of two sarcomeric proteins MYH7 and MHC in GRMD^MuStem^ muscle suggests that miR-133a and miR-222 could be involved in the remodelling of the sarcomeric assembly, thus preventing the accumulation of sarcomeric component aggregates observed in dystrophic muscle. Moreover, the pathway analysis performed to provide functional annotation based on KEGG terms (DIANA-miRPath) shows an enrichment of miR-133 in many pathways linked to ubiquitin mediated proteolysis as well as regulation of the actin cytoskeleton. Also, this indicates that miR-222 is involved in the molecular pathways linked to the cell cycle, the insulin signalling pathway and ubiquitin mediated proteolysis.

These observations corroborate our previous study [30] in which we demonstrated that systemic administration of MuStem cells greatly enhances ubiquitin-mediated protein degradation and induces insulin resistance in skeletal muscle.

## Conclusion

In the present study, we characterize the muscle expression pattern of five relevant miRNAs in the GRMD dog model. Interestingly, we define a specific global miRNA signature distinct from those found in the *mdx* mouse model but also in DMD patients. In addition, we establish that MuStem cell infusion is characterized by an up-regulation of both miR-133a and miR-222, positioning them as potential useful markers to assess the efficacy of a cell-based strategy. Further functional studies and target exploration should be carried out to improve our understanding of the links with MuStem cell-associated effects.

## Ethics approval

This study was approved by the Ethics Committee on Animal Experimentation of the Pays de la Loire Region, France, in accordance with the guidelines from the French National Research Council for the Care and Use of Laboratory Animals (Permit Number: CEEA.2012.104).

## Consent for publication

Not applicable.

## Availability of data and materials

All the data supporting the findings are contained within the manuscript.

## Additional files

Additional file 1: Figure S1. Validation of RNU6B as an internal control for study of miRNAs expression in dog muscles. The threshold cycle (CT) of RNU6B was determined in each sample and shows similar levels in healthy, GRMD, mock GRMD and GRMD^MuStem^ dogs. (TIF 239 kb) Additional file 2: Figure S2. Clinical follow-up. Clinical scores of mock GRMD dogs (---) and MuStem cell-injected dogs (—) are represented as mean ± SD. The clinical score of each GRMD dog was assessed weekly and expressed as a percentage of a theoretical healthy dog score. Limits of the MuStem cell delivery window are indicated (dashed lines). (PDF 32 kb)

## Abbreviations

DMD: Duchenne muscular dystrophy
GRMD: Golden Retriever muscular dystrophy
GRMD^MuStem^: GRMD dogs transplanted with MuStem cells
ISH: *in situ* hybridization
MHC: Myosin Heavy Chain
miRNAs: microRNAs
MyHCd: developmental Myosin Heavy Chain
