# Supplementary figures and images for: Identification in GRMD dog muscle of critical miRNAs involved in pathophysiology and effects associated with MuStem cell transplantation
Source: BMC Musculoskelet Disord. 2016 May 11;17:209. doi: 10.1186/s12891-016-1060-5 (PMC4865027; doi:10.1186/s12891-016-1060-5)

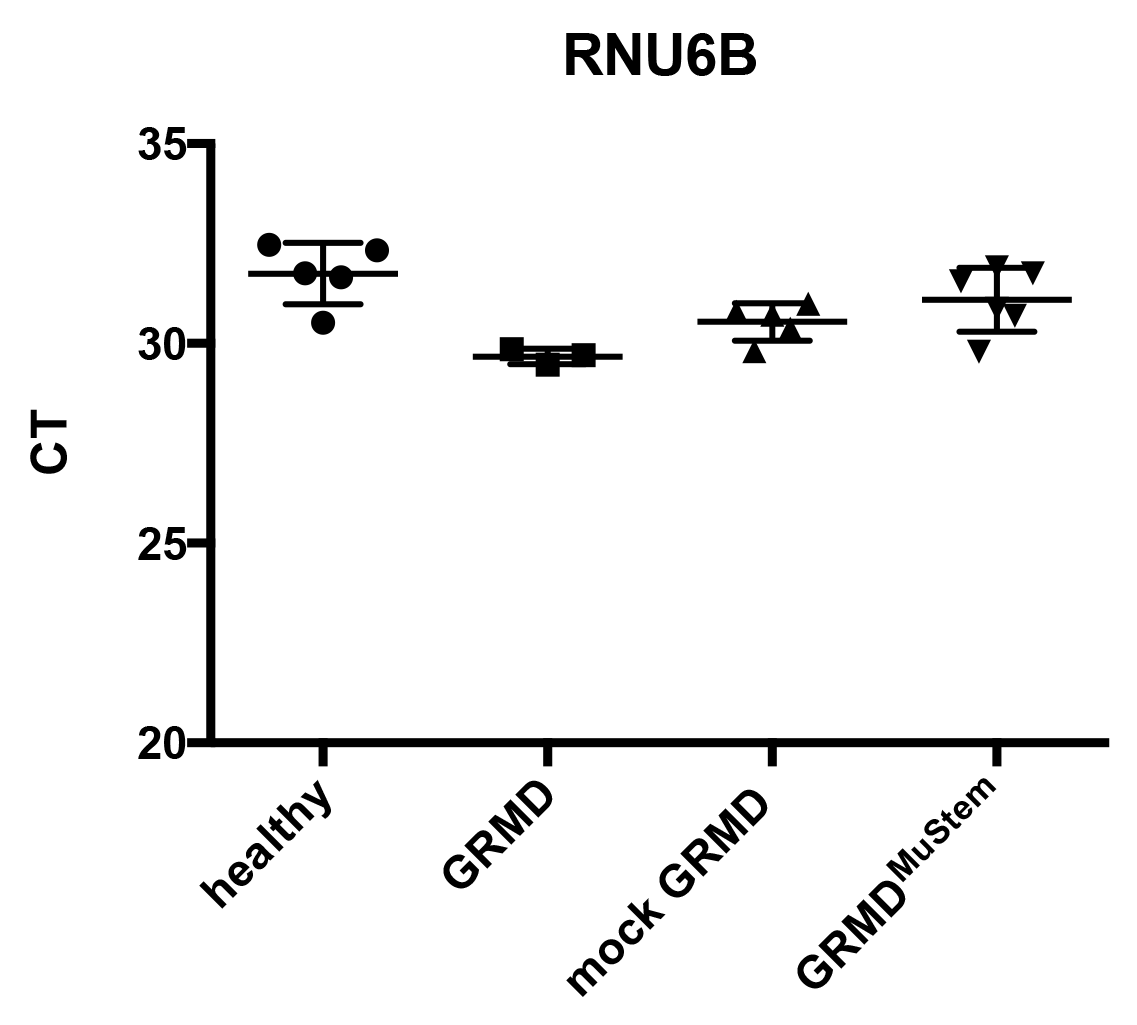

Supplement: Additional file 1: Figure S1. — Validation of RNU6B as an internal control for study of miRNAs expression in dog muscles. The threshold cycle (CT) of RNU6B was determined in each sample and shows similar levels in healthy, GRMD, mock GRMD and GRMDMuStem dogs. (TIF 239 kb) [file 12891_2016_1060_MOESM1_ESM.tif]

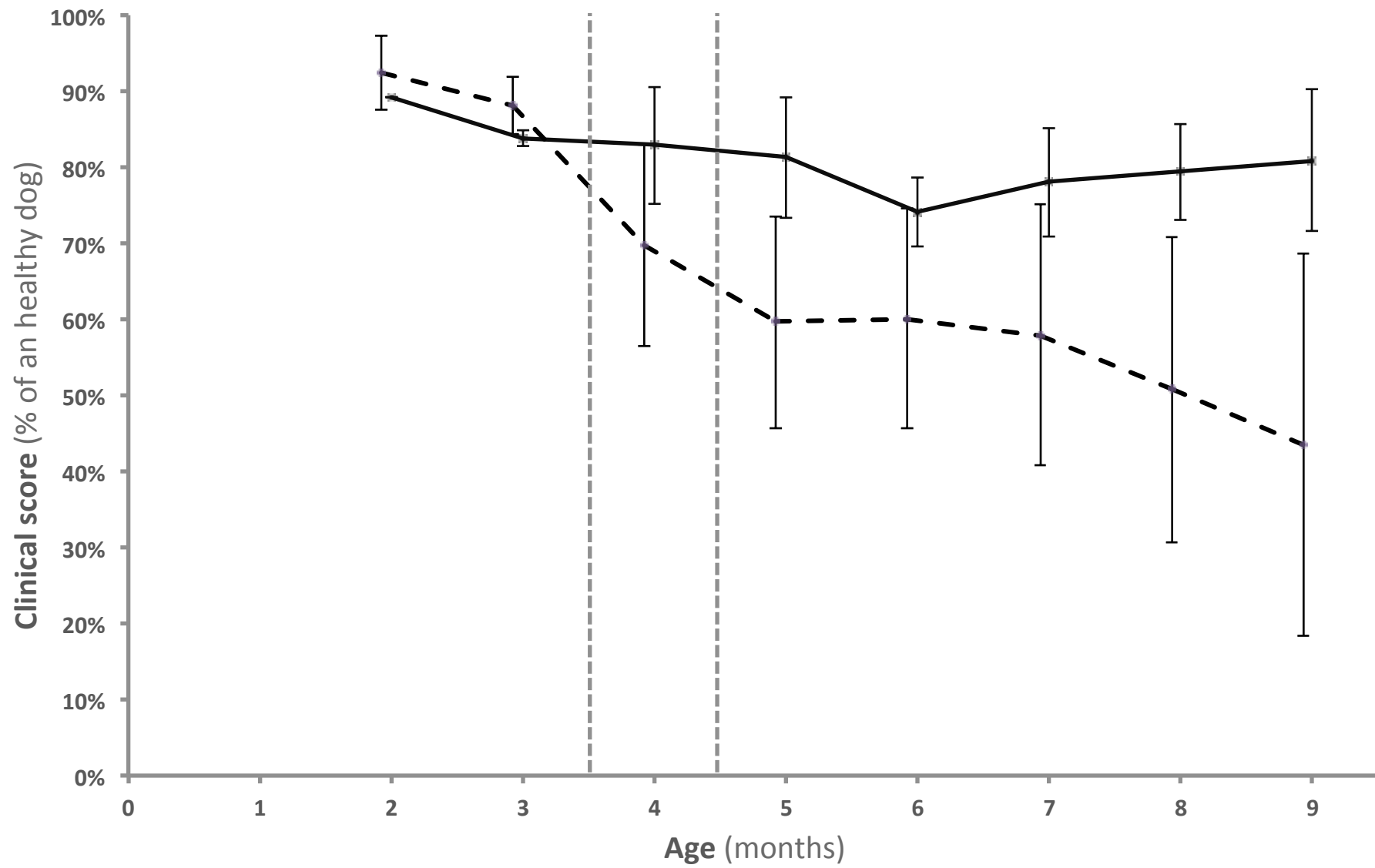

Supplement: Additional file 2: Figure S2. — Clinical follow-up. Clinical scores of mock GRMD dogs (---) and MuStem cell-injected dogs (—) are represented as mean ± SD. The clinical score of each GRMD dog was assessed weekly and expressed as a percentage of a theoretical healthy dog score. Limits of the MuStem cell delivery window are indicated (dashed lines). (PDF 32 kb) [file 12891_2016_1060_MOESM2_ESM.pdf]
